# Elucidating functional epitopes within the N-terminal region of malaria transmission blocking vaccine antigen Pfs230

**DOI:** 10.1038/s41541-021-00423-3

**Published:** 2022-01-13

**Authors:** Kazutoyo Miura, Eizo Takashima, Thao P. Pham, Bingbing Deng, Luwen Zhou, Wei-Chiao Huang, Ababacar Diouf, Yonas T. Gebremicale, Mayumi Tachibana, Tomoko Ishino, C. Richter King, Jonathan F. Lovell, Carole A. Long, Takafumi Tsuboi

**Affiliations:** 1 Laboratory of Malaria and Vector Research, National Institute of Allergy and Infectious Diseases, National Institutes of Health, 12735 Twinbrook Parkway, Rockville, MD 20852 USA; 2 Division of Malaria Research, Proteo-Science Center, Ehime University, Matsuyama, Ehime 790-8577 Japan; 3 Department of Biomedical Engineering, University at Buffalo, State University of New York, Buffalo, NY 14260 USA; 4 Division of Molecular Parasitology, Proteo-Science Center, Ehime University, Toon, Ehime 791-0295 Japan; 5 PATH’s Malaria Vaccine Initiative (MVI), Washington, DC 20001 USA; 6 Present Address: Department of Environmental Parasitology, Graduate School of Medical and Dental Sciences, Tokyo Medical and Dental University, Tokyo, Japan

**Keywords:** Peptide vaccines, Parasitic infection

## Abstract

Pfs230 is a leading malaria transmission blocking vaccine (TBV) candidate. Comprising 3135 amino acids (aa), the large size of Pfs230 necessitates the use of sub-fragments as vaccine immunogens. Therefore, determination of which regions induce functional antibody responses is essential. We previously reported that of 27 sub-fragments spanning the entire molecule, only five induced functional antibodies. A “functional” antibody is defined herein as one that inhibits *Plasmodium falciparum* parasite development in mosquitoes in a standard membrane-feeding assay (SMFA). These five sub-fragments were found within the aa 443–1274 range, and all contained aa 543–730. Here, we further pinpoint the location of epitopes within Pfs230 that are recognized by functional antibodies using antibody depletion and enrichment techniques. Functional epitopes were not found within the aa 918–1274 region. Within aa 443–917, further analysis showed the existence of functional epitopes not only within the aa 543–730 region but also outside of it. Affinity-purified antibodies using a synthetic peptide matching aa 543–588 showed activity in the SMFA. Immunization with a synthetic peptide comprising this segment, formulated either as a carrier-protein conjugate vaccine or with a liposomal vaccine adjuvant system, induced antibodies in mice that were functional in the SMFA. These findings provide key insights for Pfs230-based vaccine design and establish the feasibility for the use of synthetic peptide antigens for a malaria TBV.

## Introduction

Morbidity and mortality from malaria decreased significantly from 2000 to 2015, but progress has stalled since 2015. The World Health Organization reported 409,000 malaria-related deaths in 2019^1^. Eighty-seven countries remain endemic for malaria and 34% of malaria-endemic countries are estimated to have more cases in 2020 than in 2015^1^. Therefore, in addition to the expansion of existing anti-malarial control measures, it is critical to develop new tools, such as vaccines, to fight malaria and accelerate parasite elimination efforts. Vaccines against *Plasmodium falciparum* malaria, the most deadly malaria in humans, have attempted to target all stages of the complex life cycle. Of those vaccines, transmission blocking vaccines (TBVs) target the early stage of parasite development in the mosquitoes, the biological bottleneck of the malaria life cycle^2^. TBVs are primarily regarded as tools to accelerate parasite elimination, by breaking the cycle of transmission within communities, but could also be deployed to prevent the spread of drug-resistant parasites.

Pfs230 is a well-studied TBV candidate, and the first to reach Phase 2 clinical trials as a recombinant protein sub-fragment conjugated with *Pseudomonas aeruginosa* ExoProtein A (ClinicalTrial.gov Identifier: NCT03917654). In gametocytes (sexual-stage parasites in humans), Pfs230 is initially expressed as a 360-kDa protein, then the first 442 amino acids (aa) are cleaved and the remaining Pfs230 is exposed on the surface of gametes (sexual-stage parasites in mosquitoes that develop from gametocytes) that egress from erythrocytes in the mosquito midgut^2^. Pfs230 is a six cysteine (6-cys) family protein^3^, and is predicted to have 14 cysteine motif (CM) domains, with each domain containing 4–6 cysteine residues^4,5^. In 1987, Quakyi *et al*. identified Pfs230 as a TBV candidate using monoclonal antibodies (mAbs) raised against sexual-stage parasites^6^. In 1995, Williamson *et al*. showed that a recombinant Pfs230 protein comprising the cleaved-prodomain region (aa residues 443–588) and the first three CMs of Pfs230 (aa 589–1132) conjugated with a maltose-binding protein (r203/MBP.C) could elicit “functional” antibodies in immunized mice^7^. Here the term “functional” antibody means that it prevents oocyst formation in mosquitoes judged by a standard membrane-feeding assay (SMFA) or a direct membrane-feeding assay (DMFA), the two assays widely used to evaluate TBV candidates. For the purpose of this study, an epitope(s) that is recognized by a “functional” antibody, is referred to as a transmission-reducing epitope (TR epitope). In this study, we do not otherwise assess whether TR epitopes have any essential function in the biology of mosquito infection. Since 1995 many different Pfs230-based vaccines have been made, using a variety of expression systems^8–15^ or viral vectors^16^. However, all of them focused on aa 443–1132 (corresponding to only ~1/3 of the Pfs230 molecule) or shorter regions (e.g., the cleaved-prodomain alone, or the cleaved-prodomain plus the first two CMs). In a previous study, our group took a comprehensive approach and systematically produced one-, two- and four-CMs constructs (a total of 27 protein sub-fragments), which covered the uncleaved prodomain and all 14 CMs, to localize TR epitopes within the entire Pfs230 molecule^17^. Only five constructs, the shortest one comprising aa 543–730 and the longest one comprising aa 443–1274, induced functional antibodies in mice. The five functional constructs identified, as well as all other Pfs230 constructs tested in the preceding studies^8–16^ that induced functional antibodies, contained either the entire aa 543–730 region or a part of it. Therefore, these results suggest that all TR epitopes in the Pfs230 molecule may exist within the aa 543–730 region.

In the present work, we conducted a series of affinity depletions and purifications against different regions of Pfs230 using the polyclonal antibodies raised against the five constructs that gave rise to functional antibodies from our previous study^17^. The aim was to further assess the location of TR epitopes within the aa 443–1274 region. The results of this study suggest that TR epitope(s) do exist outside of the aa 543–730 region, but not within the aa 918–1274 (CM3 and CM4) region. In addition, we report the discovery of a 46-aa region within the cleaved-prodomain (aa 443–588) from which synthetic peptide immunogens can induce functional antibodies.

## Results

### Depletion of region-specific antibody from functional total IgGs

**Fig. 1:**
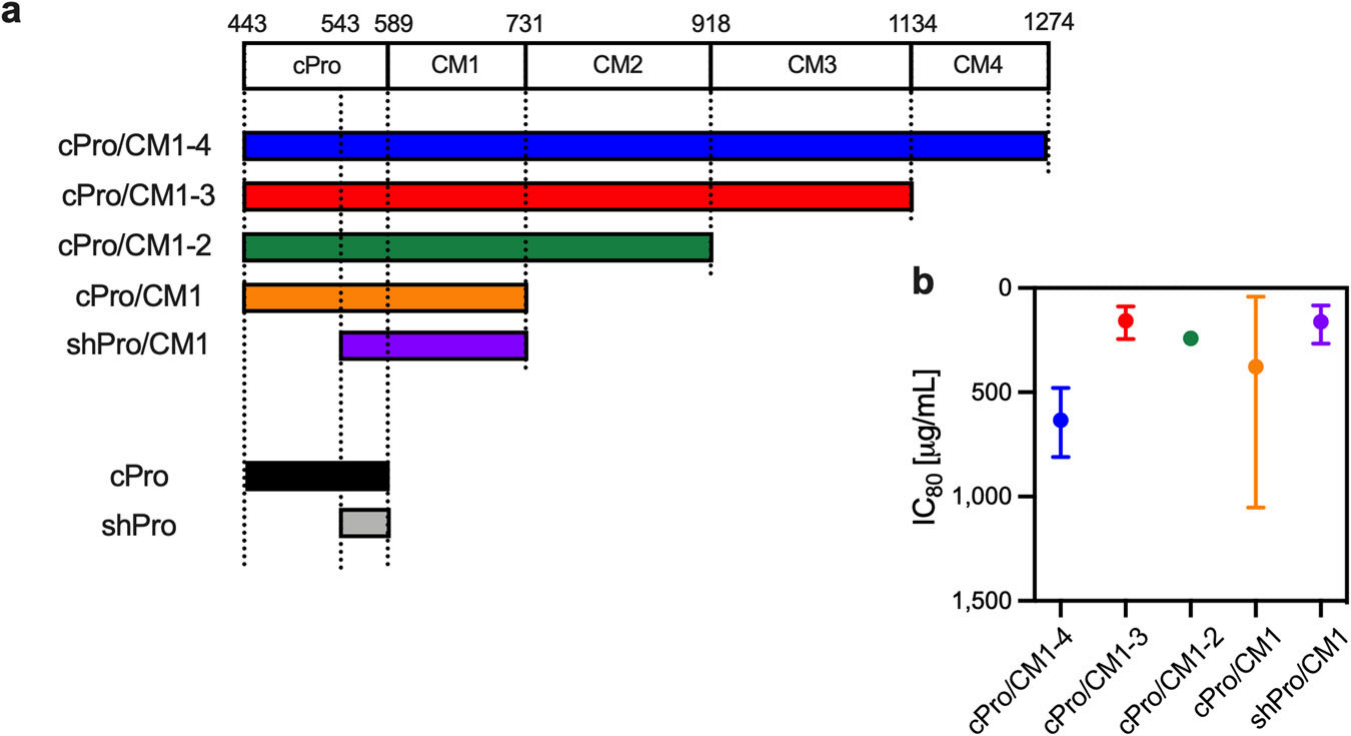
Pfs230 constructs and antibodies used in this study. **a** Pfs230 domain structure and design of constructs used in this study are shown. The numbers above Pfs230 structure bar indicate amino acid (aa) positions. CM stands for cysteine motif domain defined by Gerloff et al.^5^. Within the prodomain region (aa 22-588), only areas after cleavage site (aa 443) were included for the construct design. cPro (cleaved-prodomain) means aa 443–588 region of Pfs230, and shPro (short prodomain region) indicates aa 543–588 region. **b** Mouse antibodies against cPro/CM1-4, cPro/CM1-3, cPro/CM1-2, cPro/CM1 and shPro/CM1 generated in the previous study^17^ were tested by standard membrane-feeding assay (SMFA) at four concentrations, and total IgG concentration which gave 80% reduction in oocyst density (IC_80_) was calculated for each antibody. The IC_80_ (closed circles) and the 95% confidence interval (95%CI, error bars) are shown. For ease of comparing IC_80_ data with per cent inhibition of oocyst density (%TRA) in other figures, the *y*-axis is reversed.

In a previous study^17^, we reported that five Pfs230 constructs, TBV01 (aa 443–1274), TBV27 (aa 443–1132), TBV05 (aa 443–904), TBV12 (aa 443–730) and TBV26 (aa 543–730) elicited functional antibodies in immunized mice, while the other 22 tested fragments of Pfs230 did not. For clarity, these five constructs are renamed as “cPro/CM1-4”, “cPro/CM1-3”, “cPro/CM1-2”, “cPro/CM1” and “shPro/CM1”, respectively in this study, where “cPro” stands for the cleaved-prodomain covering aa 443–588 and shPro stands for short prodomain region covering aa 543–588 (Fig. 1a). The mouse antibodies generated against the five constructs in the previous study^17^ were tested by SMFA, and IC_80_ values, the concentration of IgG which shows an 80% reduction in oocyst density (%TRA), were estimated as 157–634 µg/mL depending on the constructs (Fig. 1b. The original SMFA results are shown in Table S1).

**Fig. 2:**
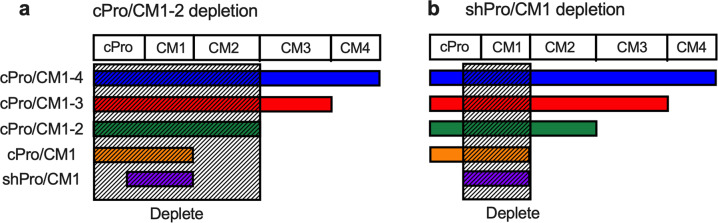
Study design for region-specific antibody depletions. **a** From the five total IgGs and anti-Glutathione S-transferase (GST) total IgG (as a negative control), cPro/CM1-2-specific antibodies were depleted using a cPro/CM1-2-coupled affinity column. The results are shown in Table 1. **b** shPro/CM1-specific antibodies were depleted using a shPro/CM1-coupled affinity column. The results are shown in Table 2.

Since multiple studies have shown that Pfs230 constructs lacking CM3 and CM4 can induce functional antibodies in rodents^9,10,12,13^, we first evaluated whether there were any TR epitopes within CM3 and CM4. The five original total IgGs were independently applied to an affinity column which was coupled with cPro/CM1-2 to deplete antibodies that bind to either cPro, CM1, or CM2 (Fig. 2a). The antibody reactivity against the cPro/CM1-2 protein in the original total IgGs (before depletion) and the flow-through fractions (depleted IgGs) were compared by ELISA, demonstrating that the efficiency of depletion was >98% for all IgG samples (Table 1). While the original IgGs showed > 94%TRA (*p* ≤ 0.001 for all) at 1.5 mg/mL in the SMFA, all cPro/CM1-2-depleted IgGs showed a loss in activity (<26%TRA, *p* > 0.435) at the same IgG concentration. We confirmed that the cPro/CM1-2-depleted IgGs from anti-cPro/CM1-4 and anti-cPro/CM1-3 total IgGs reacted with a CM3-4 recombinant protein measured by ELISA (Fig S1a) and with native Pfs230 molecule by western blot (Fig S1b). Taken together, we speculate that there are no independent TR epitopes in the CM3 and CM4 (aa 918–1274). An “independent” TR epitope in this context means that all amino acids of the TR epitope are present within CM3 and CM4.

**Table 1:** IgG depletion with cPro/CM1-2 protein.

IgG name	Antibody level^a^			Functional activity^b^					
	Total IgG	Depleted IgG		Total IgG			Depleted IgG		
	EU	EU	(%)^c^	%TRA	(95%CI)	*p*	%TRA	*(95%CI)*	*p*
cPro/CM1-4	72,657	234	(0)	95	(90–98)	0.001	−36	(−190–40)	0.458
cPro/CM1-3	80,025	1,131	(1)	100	(99–100)	0.001	10	(−103–62)	0.813
cPro/CM1-2	85,757	886	(1)	94	(89–97)	0.001	14	(−84–60)	0.741
cPro/CM1	59,753	306	(1)	99	(97–100)	0.001	26	(−55–65)	0.436
shPro/CM1	118,507	1,209	(1)	99	(97–100)	0.001	−36	(−187– 38)	0.436
GST	142	N.A.^d^		N.A.^e^			24	(−62– 65)	0.492

^a^Anti-cPro/CM1-2 ELISA units (EU).

^b^All IgGs were tested at 1.5 mg/mL in SMFA. Percent inhibition in oocyst density (%TRA), the 95% confidence interval (95%CI) and p-value are shown. The original numbers in each assay are shown in Tables S2 and S3.

^c^The proportion (%) of remaining cPro/CM1-2-specific antibodies in the depleted IgGs.

^d^Too low to determine.

^e^Anti-GST total IgG was used as a negative control.

The second region-specific antibody depletion experiment was conducted with a shPro/CM1-affinity column, as all five SMFA-positive constructs identified in the previous study^17^ contained shPro/CM1 (aa 543–730) (Fig. 2b). Similar to the previous experiment, the five original total IgGs were independently applied to the affinity column, and the efficiency of depletion was determined by ELISA (Table 2). While the efficiency of shPro/CM1-specific IgG depletion was >90% for the four samples, for one depleted IgG from the anti-shPro/CM1 total IgG, the efficiency was only 55%. Those four depleted IgGs, which showed >90% depletion efficiency, demonstrated weaker than the corresponding original total IgGs, but statistically significant activities (58, 72 and 78%TRA, respectively; *p* < 0.005 for all). The four shPro/CM1-depleted IgGs demonstrated predicted reactivities against CM2 or CM3-4 recombinant proteins by ELISA, and against native Pfs230 by western blot (Fig S2). Collectively, the results indicate a TR epitope(s) likely exists outside of shPro/CM1 region (either in the cPro or in CM2).

**Table 2:** IgG depletion with shPro/CM1 protein.

IgG name	Antibody level^a^			Functional activity^b^					
	Total IgG	Depleted IgG		Total IgG			Depleted IgG		
	EU	EU	(%)^c^	%TRA	(95%CI)	p	%TRA	(95%CI)	p
cPro/CM1-4	14,242	1,189	(8)	95	(90–98)	0.001	58	(27– 77)	0.004
cPro/CM1-3	42,980	288	(1)	100	(99–100)	0.001	72	(53–85)	0.001
cPro/CM1-2	25,974	427	(2)	94	(89–97)	0.001	78	(59–88)	0.001
cPro/CM1	34,334	239	(1)	99	(97–100)	0.001	48	(6–70)	0.032
shPro/CM1	103,414	46,286	(45)	99	(97–100)	0.001	78	(62–88)	0.001
GST	15	N.A.^d^		N.A.^e^			−3	(−81–40)	0.909

^a^Anti-shPro/CM1 ELISA units (EU).

^b^Functional activity was measured by SMFA. Percent inhibition in oocyst density (%TRA), the 95% confidence interval (95%CI) and p-value are shown. The original numbers in each assay are shown in Table S4.

^c^The proportion (%) of remaining shPro/CM1-specific antibodies in the depleted IgGs.

^d^Too low to determine.

^e^Anti-GST total IgG was used as a negative control.

### cPro-specific and shPro-specific IgGs have functional activity

**Fig. 3:**
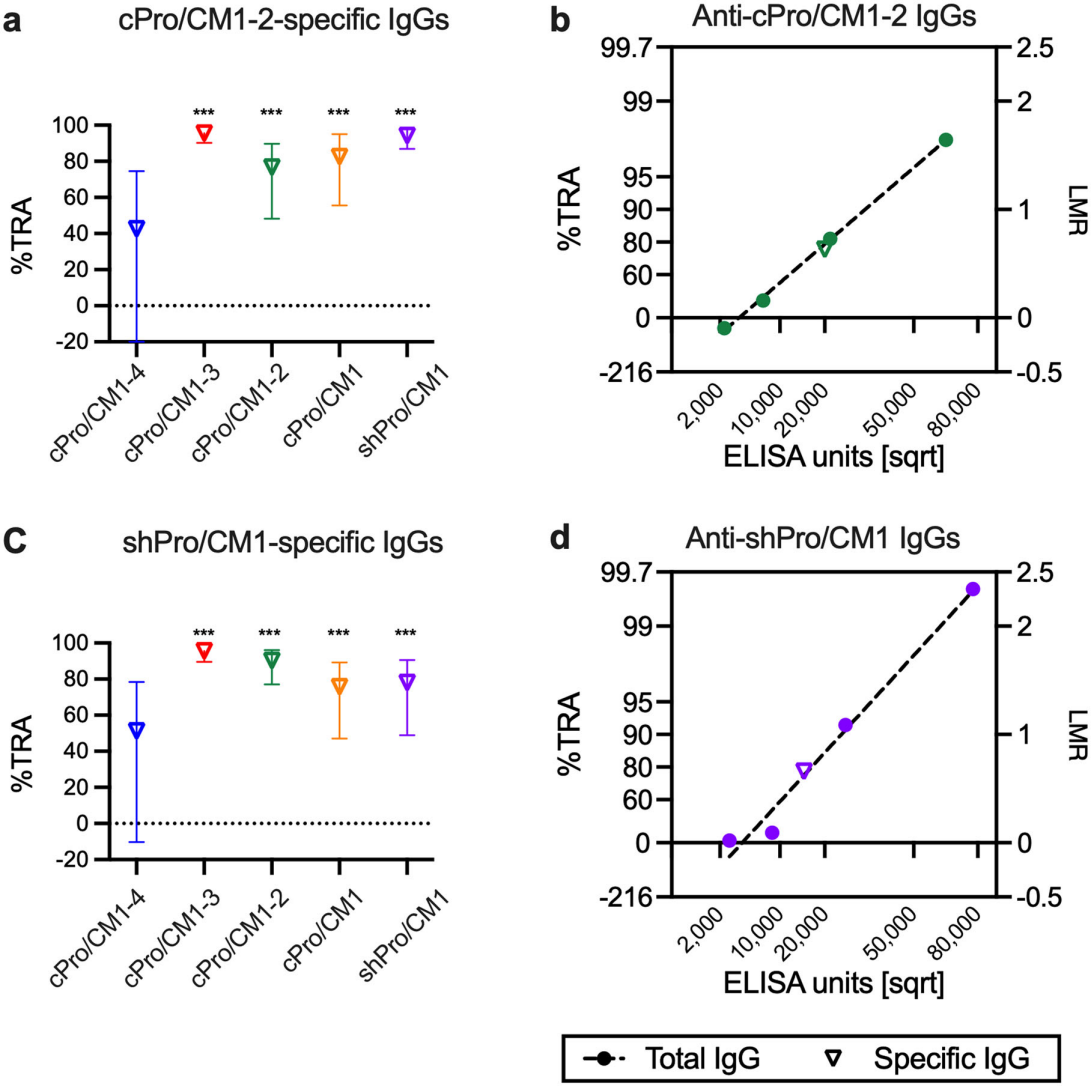
cPro/CM1-2-specific and shPro/CM1-specific antibodies are functional. a cPro/CM1-2-specific IgGs from the five total IgGs were tested at 20,000 EU in a single assay, and the %TRA and the 95%CI are shown. ***p < 0.001. **b** SMFA results of anti-cPro/CM1-2 total IgG (at four different concentrations) and cPro/CM1-2-specific IgG from the same total IgG (at 20,000 EU) are presented. Anti-cPro/CM1-2 ELISA units in a feeder (on a square root scale, sqrt) is shown on the *x*-axis, and the ratio of mean oocyst (mean oocyst density in control divided by mean in test) is plotted on a log scale (log of mean oocyst ratio, LMR, right side of *y*-axis). The associated %TRA value is shown on the left side of the *y*-axis. The dotted line represents the best estimate of the linear regression of the total IgG data (not including data point of the cPro/CM12-specific IgG). **c** shPro/CM1-specific IgG were tested at 15,000 EU in a single assay. ***p < 0.001. **d** SMFA results of anti-shPro/CM1 total IgG and the shPro/CM1-specific IgG are presented in the same fashion as **b**.

We next evaluated biological activity of antibodies that bound to the affinity column (region-specific IgGs), instead of antibodies that did not bind to the column (depleted IgGs), by SMFA. When the cPro/CM1-2-specific IgGs were tested at the highest concentration possible with the available amounts of affinity-purified IgGs in the first assay, region-specific IgGs from all five groups showed significant activities (>94%TRA, *p* < 0.001; Table S5). To determine whether those IgGs had the same %TRA at the same antibody level against cPro/CM1-2, the five region-specific IgGs were tested at 20,000 ELISA units in the second assay (Fig. 3a, Table S5). The 20,000 ELISA units were set based on the IC_80_ value of anti-cPro/CM1-2 total IgG. Except for the specific IgG from the anti-cPro/CM1-4 total IgG (43%TRA, *p* = 0.158), the region-specific IgGs from the four other groups showed 77–96%TRA (*p* < 0.002). The original anti-cPro/CM1-2 total IgG and the region-specific IgG from the same total IgG showed similar %TRA at the same ELISA units (Fig. 3b). In the next set of experiments, affinity-purified shPro/CM1-specific IgGs were also tested at IC_80_ concentration (i.e., 15,000 ELISA units) which was estimated from the original anti-shPro/CM1 total IgG (Fig. 3c, Table S6). Similar to the cPro/CM1-2-specific IgGs, the shPro/CM1-specific IgG from the anti-cPro/CM1-4 total IgG did not demonstrate significant activity (51%TRA, *p* = 0.096), while the other four region-specific IgGs did (76–96%TRA, *p* < 0.001). When the shPro/CM1-specific IgG from the anti-cPro/CM1-4 total IgG was tested at 22,158 ELISA units (the highest concentration), it showed 78%TRA (*p* = 0.002). Similar to the data shown in Fig. 3b, the affinity-purified shPro/CM1-specific IgG from the anti-shPro/CM1 total IgG showed an expected level of %TRA at 15,000 ELISA units (Fig. 3d). These results indicate that the affinity purification procedure did not alter the quality of antibodies (while there was a process loss in quantity of antibodies).

**Fig. 4:**
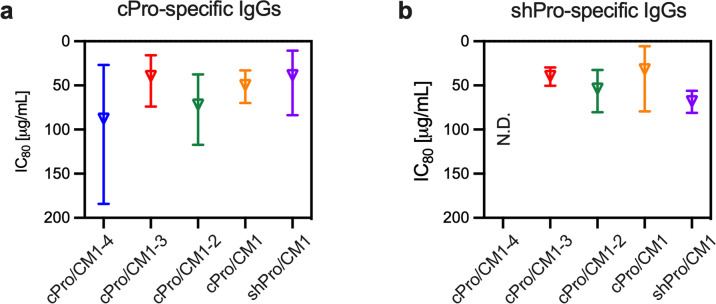
cPro- and shPro-specific antibodies are functional. **a** cPro-specific IgG from each original total IgG was tested in three independent assays at five different concentrations, then the IC_80_ (open reverse triangle) and 95%CI (error bars) were calculated. The *y*-axis is reversed as Fig. 1b. **b** shPro-specific IgGs were also tested in three SMFAs at five different concentrations, and IC_80_ (and the error range) values were calculated. N.D.: not determined, because the amount of shPro-specific IgG from anti-cPro/CM1-4 total IgG was too limited to determine IC_80_.

When the affinity-purified cPro-specific IgGs from total IgGs from the five groups were tested at the highest concentration, specific IgGs from all five groups showed >98%TRA (*p* < 0.001; Feed 1 in Table S7). Therefore, the region-specific IgGs were tested in two more independent assays at lower concentrations, and IC_80_ values for each region-specific IgG in µg/mL were calculated. The IC_80_ values were estimated from 38–88 µg/mL (Fig. 4a). Next shPro-specific IgGs which were obtained by affinity purifications from the five total IgGs were tested at the highest concentration, and four of region-specific IgGs showed >99%TRA (*p* < 0.001; Feed 1 in Table S8), while shPro-specific IgG from the anti-cPro/CM1-4 total IgG demonstrated a significant, but <80%TRA (67%TRA, *p* = 0.013). IC_80_ values were determined for the other four region-specific IgGs by performing two more assays at lower concentrations. The IC_80_ values of the four shPro-specific IgGs were estimated as 32–68 µg/mL (Fig. 4b). These results provided evidence of a TR epitope(s) in the shPro region.

### Immunization with a synthetic shPro peptide induces functional antibodies

**Fig. 5:**
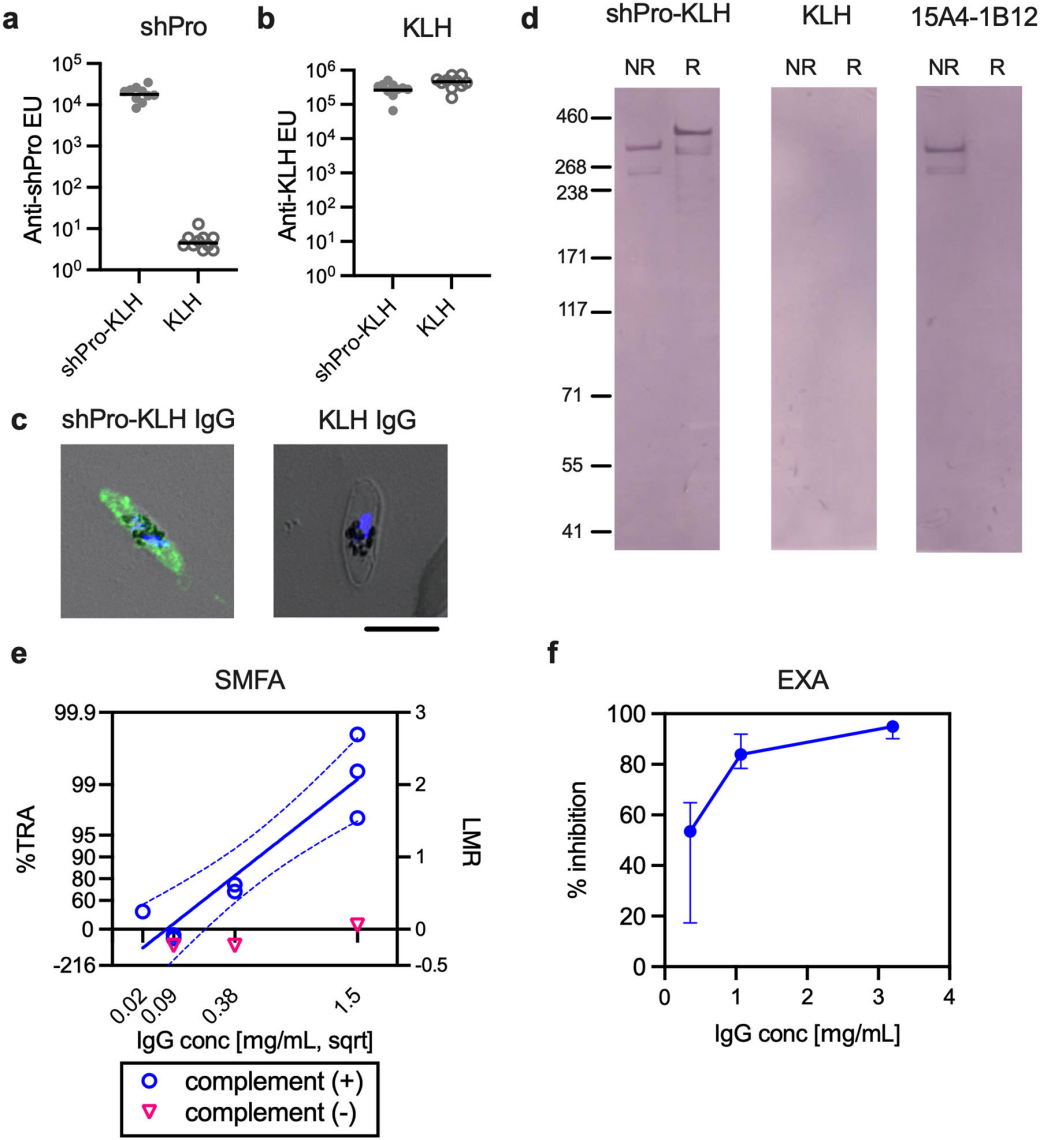
The synthetic shPro peptide (aa 543–588) induces functional antibodies. A group of ten mice were immunized with either shPro peptide conjugated with keyhole limpet haemocyanin, KLH (shPro-KLH) or KLH alone (KLH). Antibody levels in individual mice against shPro peptide (**a**) and KLH (**b**) were determined by ELISA, and the bars indicate geometric mean of the group. Reactivity of purified total IgG from each group against native Pfs230 was assessed by immunofluorescence assay (IFA, **c**) and western blotting (WB, **d**). For the IFA, *P. falciparum* NF54 stage V gametocytes were used. For the WB, gametocyte extracts were tested under reducing (R) and non-reducing (NR) conditions. The conformation-dependent mouse anti-Pfs230C1 (aa 443–731) monoclonal antibody, 15A4-1B12, was used as a positive control. **e** Anti-shPro-KLH total IgG was evaluated in three independent SMFA assays with human complement at the indicated concentrations, and one assay was conducted without complement. The solid (and dotted) line represents the best-estimate (and the 95%CI) of the linear regression of anti-shPro-KLH total IgG tested with complement. The anti-KLH total IgG tested at 1.5 mg/mL with complement showed insignificant activity (−7–30%TRA) in all three assays (Table S9). **f** Percent inhibition of anti-spr-KLH total IgG (3.2, 1.1 and 0.4 mg/mL) against anti-KLH total IgG (3.2 mg/mL) was determined by exflagellation assay (EXA). Median and range of per cent inhibitions from four assays are shown.

To directly test whether there is an independent TR epitope(s) within the shPro region, in this study, a group of ten mice were immunized intramuscularly with either a synthetic shPro peptide (aa 543–588) conjugated to keyhole limpet haemocyanin (KLH) or KLH alone (as a negative control) formulated with Montanide ISA720 on day 0 and day 28, and antisera were collected on day 42. While antisera from both groups recognized KLH equally, anti-shPro-KLH antisera specifically recognized the shPro peptide by ELISA (Fig. 5a, b). The specificity of reactivity was further evaluated by an immunofluorescence assay (IFA) with stage V mature *P. falciparum* NF54 gametocytes (Fig. 5c) and by western blotting (WB) with extracts of the mature gametocytes (Fig. 5d). In IFA, anti-shPro-KLH total IgG stained the mature gametocytes, but anti-KLH total IgG did not. For the WB, a mouse conformation-dependent anti-Pfs230 mAb, 15A4-1B12^18^, which was raised against Pfs230C1 (aa 443–731) was used as a positive control. Under non-reducing conditions, the anti-shPro-KLH total IgG and 15A4-1B12 mAb recognized proteins of the same molecular weights, while anti-KLH total IgG showed no reactivity to the gametocyte extract in the same conditions. Under reducing conditions, only the anti-shPro-KLH total IgG recognized the native Pfs230 confirming its linear epitope recognition by this IgG. When the two total IgGs were tested by SMFA, the anti-shPro-KLH total IgG showed >97%TRA (*p* < 0.001) at 1.5 mg/mL in three independent assays (Fig. 5e, Table S9), while the anti-KLH total IgG demonstrated <30%TRA (Table S9, *p* > 0.383 in all three assays). The anti-shPro-KLH total IgG showed complement- and dose-dependent activities in the SMFA. The anti-shPro-KLH total IgG was further examined by an exflagellation assay (EXA) using the anti-KLH total IgG as a negative control (Fig. 5f). The anti-shPro-KLH total IgG showed median of 54, 84 and 95% inhibition in number of exflagellation centres at 0.4, 1.1 and 3.2 mg/mL, respectively.

**Fig. 6:**
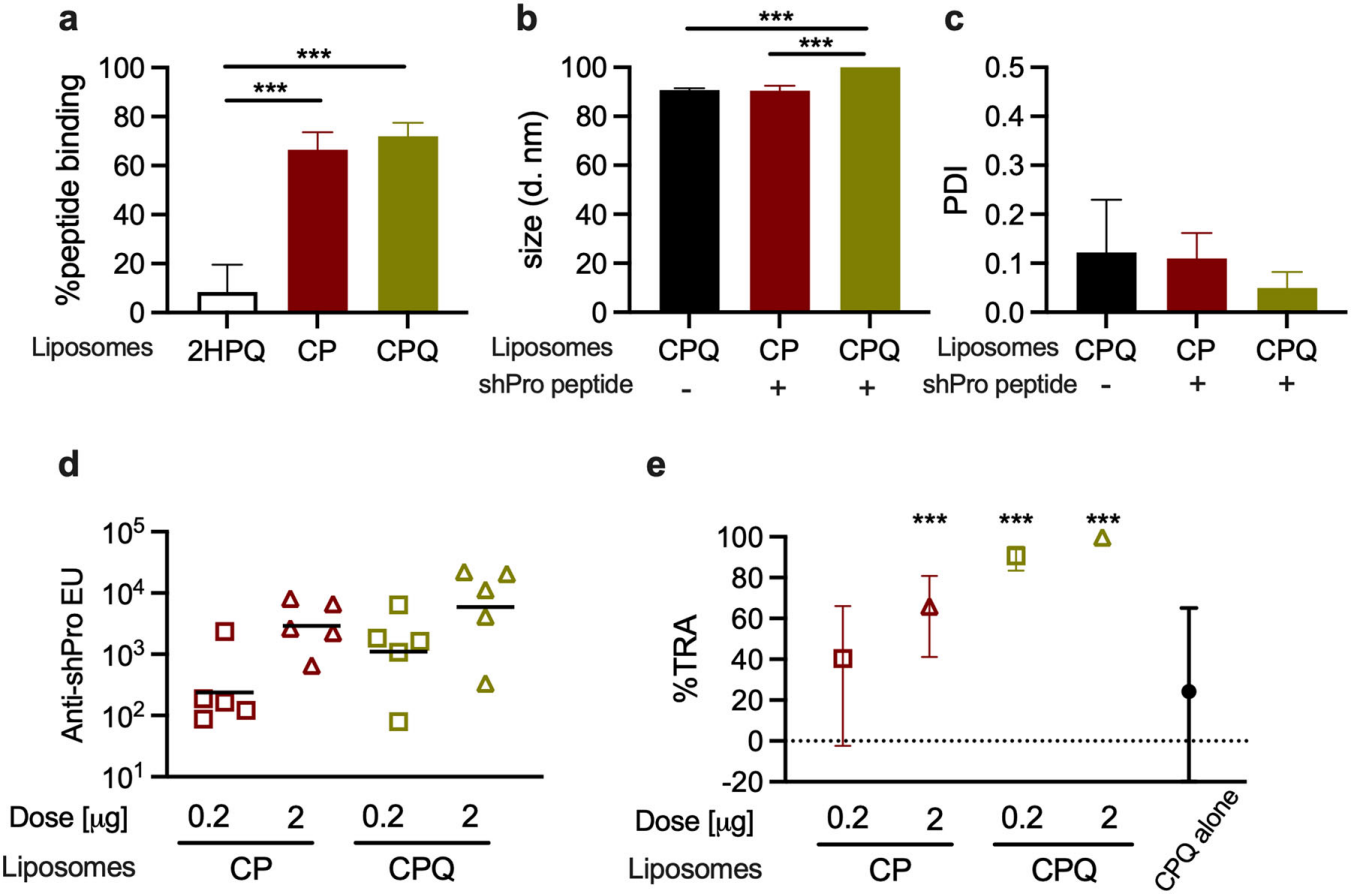
The shPro peptide formulated with CP or CPQ adjuvants elicits potent functional antibodies. **a** The percentage of peptide binding to liposomes as assessed by a microcentrifugal filtration assay. The details of 2HPQ (a negative control), CP and CPQ (CP plus QS-21) liposomes are described in the Methods section, and the mean + standard deviation (SD) for *n* = 4 experiments are shown. There was a significant difference among the three groups (*p* < 0.001 by a one-way ANOVA test). The significant differences between two groups judged by Dunn’s multiple comparisons tests are shown. ***p < 0.001. The size (**b**) and Polydispersity Index (PDI, **c**) of liposomes before and after peptide incubation. The mean + SD for *n* = 3 experiments are shown. There was a significant difference in size (*p* < 0.001), but not in PDI (*p* = 0.466). **d** A group of five mice were immunized with a 0.2 or 2 µg dose of shPro peptide (without KLH-conjugation) formulated with CP or CPQ liposomes. Antibody levels in individual mice against shPro peptide are shown with the geometric mean of the group (bars). **e** The functional activity of total IgGs was evaluated in two independent SMFA assays with human complement at 1.5 mg/mL. In addition, a purified total IgG from mice immunized with CPQ alone was tested at the same condition in SMFA. The %TRA and the 95% CI are shown. ***p < 0.001.

Encouraged by this finding, to further confirm the presence of functional epitopes within the shPro immunogen, the peptide (without KLH conjugation) was formulated with a liposome adjuvant system designed to display the antigen on its surface^19^. In the second mouse immunization study, two different liposome adjuvants, CP and CPQ, were utilized. CP contains both cobalt-porphyrin-phospholipid (CoPoP) and synthetic monophosphoryl lipid A adjuvant Hexa‐acyl Lipid A, 3‐Deacyl (PHAD‐3D6A). CPQ contains CP (above) along with the adjuvant QS-21. CP and CPQ have previously been assessed for protein-based TBV^20^ and peptide-based T-cell immunogens^21^. First, characteristics of vaccine formulations were evaluated using CP, CPQ and 2HPQ. 2HPQ is identical to CPQ but two hydrogens (“2H”) replace the cobalt, so there is no cobalt-driven association of the His-tag on the antigen with the liposome. When the shPro peptide was admixed with CP or CPQ for 3 h, >60% of the peptides bound to the liposomes, while <10% bound to 2HPQ, consistent with previous tests^21^ of this formulation (Fig. 6a). There was a slight increase of size (*p* < 0.001) and no change in size distribution (*p* = 0.890) when CPQ was incubated with the shPro peptide compared to CPQ alone (Fig. 6b and c). However, the size and size distribution between CPQ alone and the mixture of CP and the peptide were similar (*p* > 0.111 for both). Mice were immunized intramuscularly with either 0.2 or 2 µg doses of shPro peptide admixed with CP or CPQ adjuvant, on day 0 and day 21, and the level of anti-shPro antibody in each antiserum on day 42 was measured by ELISA (Fig. 6d). The higher dose of shPro peptide elicited a higher geometric mean of ELISA units in each adjuvant, and the CPQ adjuvant induced higher ELISA units than CP adjuvant at each dose. For each group, total IgG was purified from pooled sera, and tested by SMFA at 1.5 mg/mL with complement in two independent assays. The %TRA from the two feeds were 40 (*p* = 0.057), 66 (*p* < 0.001), 91 (*p* < 0.001) and 100 (*p* < 0.001)%TRA for the 0.2 µg-CP, 2 µg-CP, 0.2 µg-CPQ and 2 µg-CPQ groups, respectively (Fig. 6e, Table S10). As a negative control, purified total IgGs were collected from mice immunized with CPQ alone (without peptide) and tested at 1.5 mg/mL in the second feed. This IgG showed an insignificant activity (24%TRA, *p* = 0.490). Thus, the 46 aa shPro region is sufficient to induce functional antibodies in mice and able to do so as a synthetic peptide immunogen.

## Discussion

In follow-up to our prior survey of the entire Pfs230 molecule that suggested the presence of TR epitopes in the N-terminal region of Pfs230^17^, we here attempted to narrow their locations within the aa 443–1274 region. The results indicate that (1) CM3 and CM4 (aa 918–1274) are unlikely to contain independent TR epitopes and (2) not only shPro/CM1 (aa 543–730) but also outside of this region contains a TR epitope(s). In addition we have shown that the shPro (aa 543–588) alone is sufficient to induce transmission-reducing antibodies.

In the first part of this study, we conducted region-specific antibody-depletion experiments using two different Pfs230 sub-fragments. For the first depletion experiment with a cPro/CM1-2 recombinant protein, the depletion efficiency was high (>98%), and none of the depleted IgGs showed significant functional activity, thus it is relatively straightforward to interpret these results. CM3 and CM4 (aa 918–1274) are unlikely to contain independent TR epitopes. In the second depletion experiment, the shPro/CM1 (aa 543–730)-depleted IgG from the anti-cPro/CM1 (aa 443–730) total IgG demonstrated a weak, but statistically significant activity (48% TRA, *p* = 0.032). The results indicate that there is an independent TR epitope(s) within aa 443–542 region (the N-terminal side of cPro). The shPro/CM1-depleted IgG from the anti-cPro/CM1-2 (aa 443–904) also showed a significant activity (78%TRA, *p* < 0.001), and it is difficult to judge whether the activity in the depleted IgG came from antibodies against aa 443–542, aa 731–904 (CM2) or both. Since there was insufficient total IgG to perform an additional depletion experiment with a cPro/CM1-coupled affinity column, we could not examine the effect of anti-CM2-specific antibodies in this study. Nonetheless, the positive SMFA results with all five shPro/CM1-depleted IgGs strongly indicate that there is an independent TR epitope(s) outside of the shPro/CM1 region.

We next performed complementary SMFA experiments using region-specific IgGs. Since immunizations with cPro/CM1-2, shPro/CM1 or cPro recombinant proteins induced functional antibodies previously^10,17^, it was hypothesized that affinity-purified cPro/CM1-2-, shPro/CM1- or cPro-specific IgGs would maintain significant activities. As anticipated, those region-specific IgGs demonstrated significant function in the SMFA. Of note, the quality of the region-specific IgGs from the anti-cPro/CM1-4 total IgGs were generally lower than the other region-specific IgGs from the other four total IgGs. When cPro/CM1-2- and shPro/CM1-specific IgGs from the five total IgGs were tested at the same ELISA units, the region-specific IgGs from anti-cPro/CM1-4 total IgG showed insignificant inhibitions, while the other four region-specific IgGs showed significant inhibitions. The results suggest that inclusion of an extra region in a Pfs230-based vaccine may be undesirable for three reasons; (1) it may induce more antibody responses against non-TR epitopes, (2) lower the quality of antibody against TR epitopes and/or (3) an addition of an extra region may alter overall folding of the construct. Future vaccine research should carefully evaluate whether there is any benefit to including an extra region that does not contain TR epitopes. To our surprise, all affinity-purified shPro-specific IgGs from the five total IgGs showed significant activity. The results suggest that at least some of the functional antibodies in the total IgGs recognized the shPro region. Two recent crystallography studies have identified specific epitopes of two functional anti-Pfs230 mAbs, 4F12^22^ and LMIV230-01^23^. The 4F12 was selected based on its reactivity against a Pfs230D1M (aa 542–736) protein, and the LMIV230-01 mAb was isolated from volunteers immunized with the same protein. The two mAbs recognize amino acids both in shPro and CM1 (six and ten amino acids in shPro and CM1, respectively, for the 4F12 mAb, and 14 and 13 aa for the LMIV230-01 mAb) as shown in Fig S3. The two studies suggest that a part of “shPro-specific” antibodies may have some affinity to the shPro and the CM1 regions. Consequently, at this stage of our analysis, we cannot determine whether the functional activity seen by the “shPro-specific” IgGs are due to antibodies that bind to both shPro and CM1 region or those that exclusively bind to shPro region, thus complicating the interpretation of our results.

To investigate whether there is an independent TR epitope(s) in the shPro (i.e., whether antibodies that are elicited by epitopes exclusively within the shPro can exhibit a functional activity), we conducted mouse immunization studies with vaccine candidates based on synthetic shPro peptides. The shPro-KLH conjugate induced functional antibodies, demonstrating that the shPro alone contains a TR epitope(s). The inhibition of anti-shPro-KLH antibodies was complement-dependent, and the antibodies also reduced exflagellation as determined by EXA. These characteristics are similar to the mouse anti-Pfs230C1 (aa 443–731) antibodies^13^.

Peptide-based vaccines have several advantages over the recombinant protein-based vaccines, such as ease of manufacture, ease of characterization and ease of incorporation into a multivalent vaccine. More than 600 clinical trials with peptide antigens have been performed as of 2020^24^. On the other hand, disadvantages of peptide-based vaccines include that they provide a limited number of epitopes, they have difficulty to faithfully represent three-dimensional protein antigen structures, and they tend to have low immunogenicity.^24^ Two other notable studies have suggested that peptide-based *P. falciparum* TBVs may be able to induce functional antibodies in rodents, one being a PfHAP2 peptide (18 aa)^25^ and the other being a Pfs47 peptide (58 aa)^26^. To be confident of our own initial results (Fig. 5), we conducted a second independent mouse immunization study that involved a re-synthesis of the shPro peptide (without KLH conjugation) and an alternative formulation strategy using the CP and CPQ liposome adjuvant system (Fig. 6), which has recently entered human clinical testing in a SARS-CoV-2 vaccine (ClinicalTrial.gov Identifier: NCT04783311). When CPQ was used as an adjuvant, a 0.2 µg dose of the shPro peptide was sufficient to induce functional antibodies (91%TRA, *p* < 0.001 at 1.5 mg/mL), indicating the lower immunogenicity of the peptide-vaccine could be overcome by optimizing the vaccine formulation using a particulate antigen presentation system. The shPro (aa 543–588) is appealing for vaccine development as it contains no cysteine residues. The second advantage of shPro as a vaccine candidate is that it is highly conserved. The only polymorphism reported is P550S with minor allele frequency of <0.1% in MalariaGEN (https://www.malariagen.net/) which includes >2,500 Pfs230 sequences from >20 countries. ELISA analysis showed that mouse antibodies raised against shPro-KLH or shPro (shown in Figs. 5 or 6) reacted almost equally to the original shPro peptide and a mutant shPro peptide which contains the polymorphism (Fig S4). In addition, no significant amino acid sequence similarity with human proteins was found by a BLAST search (only similarities identified were the shPro region of Pfs230 in *P. falciparum*, or Pfs230 orthologs in chimpanzee or gorilla malaria parasites). The identification of this short peptide-based TBV candidate should open a new avenue for future Pfs230-based TBV development. It may be possible to learn more about the potency of epitopes located in the shPro region as additional mAbs to Pfs230 become available, and a head-to-head comparison study with shPro-based and other preceding Pfs230-based vaccine candidates is awaited.

While the region-specific antibody depletion/affinity purification used in our study is a useful tool for TR epitope identification, there are several caveats to mention with these results. First, as discussed above, if there are antibodies that recognize multiple regions (e.g., shPro and CM1), the results of SMFA with the “region-specific”-depleted- or -purified antibodies need to be evaluated with caution. Second, it is difficult to prove that a structure of a specific region in a recombinant protein is identical to that of the same region in another recombinant protein or in a native Pfs230 protein expressed on the surface of parasites. The third potential issue involves the binding capacity of an affinity column. For example, the shPro/CM1-depletion efficiency from the anti-shPro/CM1 total IgG was low (45%), and more cycles of depletion might increase the efficiency. However, as there was a process loss in each cycle, we decided to stop after 2-cycles of depletion in this study. Lastly, the intrinsic error of the SMFA tends to be large, especially when low %TRA is observed^27^. Therefore, it is possible that an insignificant low activity could be a weak, but true activity (e.g., CM3 might contains a very weak TR epitope(s)). Despite these limitations, this study indicates that there are TR epitope(s) outside of shPro/CM1 (aa 543–730) but within cPro/CM1-2 (aa 443–918). In addition, it has been proven that the shPro (aa 543–588) comprises a TR epitope and can be used directly as a synthetic TBV peptide immunogen to elicit functional antibodies. These findings should be useful for future Pfs230-based TBV research and development.

## Methods

### *P. falciparum* Pfs230 recombinant proteins, peptides and liposome adjuvants

The details of protein expression and purification methods for the three recombinant proteins (shPro/CM1, cPro/CM1-2 and cPro; Fig. 1), which were used for region-specific affinity depletion/purification, and one recombinant protein (CM3-4; Fig S1), which was used for ELISA, were published previously^10,17^. In brief, a sequence of Pfs230 (PF3D7_0209000) was obtained from the PlasmoDB database, and the recombinant proteins were expressed using a eukaryotic wheat germ cell-free expression system (WGCFS; CellFree Sciences, Matsuyama, Japan), and Ni-affinity-purification was performed.

The shPro peptide (aa 543–588 of Pfs230 plus one cysteine at the N-terminus for conjugation), which were used for shPro-specific depletion/purification, ELISA and the first mouse immunization study conducted at the Laboratory of Malaria and Vector Research (LMVR) of the National Institute of Allergy and Infectious Disease (NIAID), NIH, were prepared at Research Technologies Branch (RTB), NIAID. The purity of the peptide was assessed as >99.9% by HPLC analysis (Fig S5a). The shPro peptide was conjugated with keyhole limpet haemocyanin (KLH) at RTB, NIAID. As a negative control for the first mouse immunization study, KLH protein was purchased from Sigma (St. Louis, MO, USA). The other shPro peptide (aa 543–588 of Pfs230 plus six histidine residues at the C-terminus) was used for the second mouse immunization study at the State University of New York (SUNY) at Buffalo was purchased from WatsonBio Sciences (Houston, TX, USA), and the purity was 95.1% by HPLC analysis (Fig S5b).

The following lipids were used to prepare adjuvants for the second immunization study: CoPoP and PoP (produced as previously described^28^), 1,2‐Dioleoyl‐sn‐glycero‐3‐phosphocholine (DOPC, Avanti, Alabaster, AL, USA, Cat # 850 375), cholesterol (PhytoChol, Wilshire Technologies, Princeton, NJ, USA, Cat # 57-88-5), synthetic monophosphoryl lipid A Hexa‐acyl Lipid A, 3‐Deacyl (PHAD‐3D6A, Avanti Cat # 699 855) and QS‐21 (Desert King, San Diego, CA, USA).

### Mouse anti-Pfs230 antibodies

For the region-specific affinity depletion/purification studies, Protein G purified mouse anti-Pfs230 antibodies generated from a previous study^17^ were utilized. Briefly, a group of seven ICR mice were immunized with 20 µg/dose of recombinant protein with Freund’s complete (on day 0) or Freund’s incomplete adjuvant (on day 21), sera were collected on day 42, then total IgGs were prepared by Protein G using a pooled antisera for each group. The characteristics of each anti-Pfs230 polyclonal antibody were determined by western blotting (WB), immunofluorescence assay (IFA) and SMFA, and the results were reported previously^17^.

In this study, anti-shPro-KLH and anti-KLH antisera were newly generated from the first immunization experiment. A group of ten CD-1 female mice (7–9 weeks old) were immunized with 25 µg/dose of shPro-KLH protein formulated with Montanide ISA720 (Seppic, Fairfield, NJ, USA) on days 0 and 28 by intramuscular injection, and the serum samples were collected on day 42. As a negative control, another ten mice were immunized with 25 µg/dose of KLH protein. This study was conducted at LMVR, NIAID in compliance with the Animal Welfare Act regulations in the Guide for Care and Use of Laboratory Animals and reviewed and approved by NIAID’s Animal Care and Use Committee (LMVR10E).

The second mouse immunization study was carried out at SUNY under protocols approved by the University at Buffalo Institutional Animal Care and Use Committee (IACUC). A group of five CD-1 female mice (5–6 weeks old) were immunized with 0.2 or 2 µg/dose of shPro peptide formulated with CP or CPQ adjuvants on days 0 and 21 by intramuscular injection, and the serum samples were collected on day 42. As a negative control, a group of six CD-1 female mice (5–6 weeks old) were immunized with CPQ adjuvant alone in the same way, and the day 42 sera were collected.

For SMFA, total IgG was purified from pooled antisera for each group using a protein G column (GE Healthcare, Pittsburgh, PA, USA) according to the manufacturer’s instructions.

### Region-specific affinity depletion/purification

The basic methodology for region-specific affinity depletion/purification has been published elsewhere^29^. In brief, a target protein or peptide were immobilized on NHS-Active Sepharose 4 Fast Flow (GH Healthcare) by amine coupling according to the manufacturer’s instructions. Total IgGs (~4 mg each) were loaded onto the column, and the flow-through fraction was collected. The binding fraction was eluted using a low pH elution buffer and immediately neutralized by pH 9.0 Tris. The eluted fractions from the affinity purification processes were buffer exchanged with 1× Phosphate-buffered saline (PBS), and concentrated to ~100 µL of final products (“specific” IgG). To increase the efficiency of depletion, the flow-through fraction was reapplied to the same column. The flow-through fraction from the second affinity purification process was buffer exchanged with 1× PBS, and concentrated to 4 mg/mL (“depleted” IgGs).

### ELISA, SMFA and exflagellation assay (EXA)

The details of the ELISA method have been published elsewhere^30^. Per cent remaining in depleted IgG was calculated as 100 × (ELISA units in 1 mg/mL of depleted IgG)/(ELISA units in 1 mg/mL of original total IgG).

The standardized methodology for performing the SMFA has been described previously^27^. Briefly, 16–18 day old gametocyte cultures of the *P. falciparum* NF54 line were mixed with test IgGs at indicated concentration, and the final mixture was immediately fed to ~50 female *Anopheles stephensi* mosquitoes through a membrane-feeding apparatus. All feeding experiments were performed with human complement (i.e., with 31% v/v of non-heat inactivated human serum) except one set of feeds shown in Fig. 5e (Feed 3 in Table S9). Mosquitoes were kept for 8 days and dissected (*n* = 20 per group) to enumerate the oocysts in the midgut from mosquitoes with any eggs in their ovaries at the time of dissection.

The basic methodology for EXA was published before^13^. In brief, a mixture of mature gametocytes and a test total IgG was incubated at 19 °C for 18 min, and then loaded to a cellometer (Nexcelom, Lawrence, MA, USA) to determine the number of exflagellation centres. Anti-shPro-KLH IgG was tested at 3.2, 1.1 and 0.4 mg/mL, and anti-KLH IgG was at 3.2 mg/mL in four assays. Per cent inhibition of anti-shPro-KLH IgG at each concentration was calculated against anti-KLH IgG in each assay, then the median (and range) per cent inhibition from the four assays is reported.

The human serum and red blood cells used for the gametocyte cultures, SMFA and EXA were purchased from Interstate Blood Bank (Memphis, TN, USA).

### Western blotting (WB) and immunofluorescence assay (IFA)

The basic methodologies of WB and IFA have been published elsewhere^13,17^. For both assays, mature gametocytes from *P. falciparum* NF54 line, which were cultured for SMFA, were utilized. For WB, extract from 10^6^ parasites/lane were loaded and total IgGs (either anti-shPro-KLH or anti-KLH IgGs) were tested at 5 µg/mL. For IFA, the total IgGs were tested at 1 µg/mL.

### Liposome preparation, characterization, and the vaccine formulations

Ethanol and PBS were pre-heated to 50 °C. Lipids (DOPC, Cholesterol, CoPoP, and 3D6A-PHAD) were weighed into each vial and dissolved in 1 mL pre-heated ethanol. The lipid-ethanol solution was briefly sonicated to break up large particles. The lipid-ethanol solution was pre-heated to 50 °C for 10 min. Four mL of PBS was added and the solution was swirled and maintained at 50 °C for another 10 min. The solution was then briefly sonicated to break up any large particles followed by nitrogen-pressurized extrusion ten times through stacked 200 nm, 100 nm and 80 nm polycarbonate filters at 50 °C. Following extrusion, liposomes were dialysed in 500 mL of PBS with one buffer change after 12 hr and a second dialysis against PBS for an additional 4 hr. Liposomes were filtered using a 0.22 μm filter. The CoPoP/ PHAD-3D6A (“CP”) liposome formulation had a mass ratio of [DOPC:Cholesterol:CoPoP:PHAD-3D6A] of [20:5:1:0.4]. The CoPoP/PHAD-3D6A/QS‐21 (“CPQ”) liposomes had a formulation of [DOPC:CHOL:CoPoP:PHAD-3D6A:QS‐21] of [20:5:1:0.4:0.4]. PoP/PHAD-3D6A/QS-21 liposomes (“2HPQ”) had a formulation of [DOPC:CHOL:PoP: PHAD-3D6A:QS‐21] of [20:5:1:0.4:0.4]. For liposomes containing QS‐21, QS‐21 (1 mg/mL) was added to the liposomes after formation at an equal mass ratio as PHAD-3D6A, and the final liposome concentration was adjusted to 320 µg/mL of CoPoP or PoP.

For the characterization of liposome/shPro peptide formulation was carried out by incubating peptides and liposomes with a 1:4 mass ratio of peptide:CoPoP for 3 hr at room temperature (RT). Following incubation, the sample was subjected to microcentrifugal filtration (PALL Nanosep, AZ, USA, Cat # 29300,) and the peptide in the filtrate was assessed by micro BCA (Thermo Fisher Scientific, Waltham, MA, USA, Cat # 23235). The samples were spun at 1200 g for 1 hr at RT, and the filtrate was collected to measure protein concentration by micro BCA. Liposome size and polydispersity index (PDI) were determined by dynamic light scattering (NanoBrook 90 plus PALS, Brookhaven Instruments Corporation, Holtsville, NY, USA) after 200-fold dilution in PBS.

The vaccine formulations used for the second mouse immunization study were prepared by incubating the shPro peptide at a concentration of 80 µg/mL with liposomes (with a CoPoP concentration of 320 µg/mL) for 3 hr at RT prior to final dilution and immunization without any further purification. Thus, the immunization doses (0.2 and 2 µg/dose of peptides) refer to the total amount of peptides in the formulations (i.e., both bound and unbound to the liposomes).

### Statistical analysis

The per cent inhibition in oocyst density (%TRA), the 95% confidence interval (95%CI) and *p*-value from single or multiple feeds were calculated using a zero-inflated negative binomial (ZINB) model as before^31^. IC_80_, IgG concentration which gives 80%TRA, was calculated from a plot where IgG concentration was transformed in a square root scale and %TRA was transformed in a Log of mean oocyst ratio (LMR) scale, as described previously^32^. To compare per cent of peptide binding, size and PDI among three groups, one-way ANOVA followed by Dunn’s multiple comparisons tests were utilized.

All statistical tests were performed in JMP13 (SAS Institute, Cary, NC, USA), Prism 8 (GraphPad Software, La Jolla, CA, USA) or R (version 3.5.3, The R Foundation for Statistical Computing) and *p*-values < 0.05 are considered significant.

### Reporting summary

Further information on research design is available in the Nature Research Reporting Summary linked to this article.

## Supplementary information

REPORTING SUMMARY

Supplementary Tables and Figures

## Data Availability

The data that support the findings of this study are available from the corresponding author upon reasonable request. The original SMFA data underlying all figures and tables are provided in Supplemental Tables 1–10, and uncropped western blot images for Fig. 5d are seen in Supplemental Fig 6.
