# Supplementary material for: Elucidating functional epitopes within the N-terminal region of malaria transmission blocking vaccine antigen Pfs230
Source: NPJ Vaccines. 2022 Jan 13;7:4. doi: 10.1038/s41541-021-00423-3 (PMC8758780; doi:10.1038/s41541-021-00423-3)
Supplement: Supplementary file 1 — REPORTING SUMMARY [file 41541_2021_423_MOESM1_ESM.pdf]

nature research

Corresponding author(s): Takafumi Tsuboi

Reporting Summary

Nature Research wishes to improve the reproducibility of the work that we publish. This form provides structure for consistency and transparency in reporting. For further information on Nature Research policies, see Authors & Referees and the Editorial Policy Checklist.

Statistical parameters

When statistical analyses are reported, confirm that the following items are present in the relevant location (e.g. figure legend, table legend, main text, or Methods section).

n/a	Confirmed	
☐	☒	The exact sample size (n) for each experimental group/condition, given as a discrete number and unit of measurement
☐	☒	An indication of whether measurements were taken from distinct samples or whether the same sample was measured repeatedly
☐	☒	The statistical test(s) used AND whether they are one- or two-sided<br>*Only common tests should be described solely by name; describe more complex techniques in the Methods section.*
☒	☐	A description of all covariates tested
☐	☒	A description of any assumptions or corrections, such as tests of normality and adjustment for multiple comparisons
☐	☒	A full description of the statistics including central tendency (e.g. means) or other basic estimates (e.g. regression coefficient) AND variation (e.g. standard deviation) or associated estimates of uncertainty (e.g. confidence intervals)
☐	☒	For null hypothesis testing, the test statistic (e.g. F, t, r) with confidence intervals, effect sizes, degrees of freedom and P value noted<br>*Give P values as exact values whenever suitable.*
☒	☐	For Bayesian analysis, information on the choice of priors and Markov chain Monte Carlo settings
☒	☐	For hierarchical and complex designs, identification of the appropriate level for tests and full reporting of outcomes
☒	☐	Estimates of effect sizes (e.g. Cohen's d, Pearson's r), indicating how they were calculated
☐	☒	Clearly defined error bars<br>*State explicitly what error bars represent (e.g. SD, SE, CI)*

Our web collection on statistics for biologists may be useful.

Software and code

Policy information about availability of computer code

Data collection	No code was used
Data analysis	All statistical tests were performed in JMP13 (SAS Institute, Cary, NC, USA), Prism 8 (GraphPad Software, La Jolla, CA, USA) or R (version 3.5.3, The R Foundation for Statistical Computing).

For manuscripts utilizing custom algorithms or software that are central to the research but not yet described in published literature, software must be made available to editors/reviewers upon request. We strongly encourage code deposition in a community repository (e.g. GitHub). See the Nature Research guidelines for submitting code & software for further information.

Data

Policy information about availability of data

All manuscripts must include a data availability statement. This statement should provide the following information, where applicable:

- Accession codes, unique identifiers, or web links for publicly available datasets
- A list of figures that have associated raw data
- A description of any restrictions on data availability

The data that support the findings of this study are available from the corresponding author upon reasonable request. The original SMFA data underlying all figures and tables are provided in Supplemental Tables 1 - 10.

Field-specific reporting

Please select the best fit for your research. If you are not sure, read the appropriate sections before making your selection.

☒ Life sciences ☐ Behavioural & social sciences

For a reference copy of the document with all sections, see nature.com/authors/policies/ReportingSummary-flat.pdf

Life sciences

Study design

All studies must disclose on these points even when the disclosure is negative.

Sample size	The animal immunization studies were designed to answer a qualitative question (i.e., whether a new peptide antigen can induce functional antibodies in mice). Therefore, no power calculation is required.
Data exclusions	No data were excluded.
Replication	Except Fig 6 a and b where we measured characterization of vaccine formulations, we performed two independent assays to claim a "statistically significant" effect, and all original numbers are reported in the supplemental tables.
Randomization	The mice were assigned to immunization groups randomly.
Blinding	This manuscript only reports results of mouse immunization studies. Therefore, as in the most of published studies, we did not perform any blinding process .

Materials & experimental systems

Policy information about availability of materials

n/a	Involved in the study	
☐	☒	Unique materials
☒	☐	Antibodies
☒	☐	Eukaryotic cell lines
☒	☐	Research animals
☒	☐	Human research participants

Unique materials

Obtaining unique materials	The following lipids were used to prepare adjuvants; 1,2-Dioleoyl-sn-glycero-3-phosphocholine (DOPC, Avanti, Alabaster, AL, USA, Cat # 850375), cholesterol (PhytoChol, Wilshire Technologies, Princeton, NJ, USA, Cat # 57-88-5), synthetic monophosphoryl lipid A Hexa-acyl Lipid A, 3-Deacyl (PHAD-3D6A, Avanti Cat # 699855) and QS-21 (Desert King, San Diego, CA, USA)

Method-specific reporting

n/a	Involved in the study	
☒	☐	ChIP-seq
☒	☐	Flow cytometry
☒	☐	Magnetic resonance imaging
