# Supplementary material for: Elucidating functional epitopes within the N-terminal region of malaria transmission blocking vaccine antigen Pfs230
Source: NPJ Vaccines. 2022 Jan 13;7:4. doi: 10.1038/s41541-021-00423-3 (PMC8758780; doi:10.1038/s41541-021-00423-3)
Supplement: Supplementary file 2 — Supplementary Tables and Figures [file 41541_2021_423_MOESM2_ESM.pdf]

Supplemental information

Elucidating functional epitopes within the N-terminal region of malaria transmission blocking antigen Pfs230

Kazutoyo Miura, Eizo Takashima, Thao P. Pham, Bingbing Deng, Luwen Zhou, Wei-Chiao Huang, Ababacar Diouf, Yonas T. Gebremicale, Mayumi Tachibana, Tomoko Ishino, C. Richter King, Jonathan F. Lovell, Carole A. Long, Takafumi Tsuboi

Supplementary Table 1: SMFA data of original total IgG (Fig 1b)

IgG name	IgG Conc. [μg/mL]	Mean oocyst	%TRA[a]		
			%TRA	95%CI	p-value
Control IgG[b]	*750*	*33.1*			
cPro/CM1-4	750	5.3	84.1	64.3 to 92.9	0.001
	250	16.5	50.2	-9.9 to 77.7	0.085
	83	26.8	19.1	-82.1 to 63.9	0.612
	28	35.6	-7.6	-150.3 to 52.8	0.844
cPro/CM1-3	750	0.2	99.5	98.3 to 100	0.001
	250	2.7	92.0	81.7 to 96.7	0.001
	83	23.2	30.0	-56.9 to 70.2	0.375
	28	30.3	8.5	-112.2 to 61.3	0.860
cPro/CM1-2	750	0.8	97.7	94.1 to 99.5	0.001
	250	6.2	81.4	56.1 to 92.2	0.001
	83	22.9	30.9	-55.9 to 70.2	0.384
	28	41.2	-24.5	-179.4 to 45.6	0.579
cPro/CM1	750	2.6	92.1	81.6 to 97.0	0.001
	250	15.8	52.3	-14.4 to 79.1	0.083
	83	29.6	10.4	-106.1 to 58.8	0.796
	28	18.2	45.1	-25.4 to 75.1	0.154
shPro/CM1	750	0.2	99.5	98.7 to 99.9	0.001
	250	2.7	91.8	81.0 to 96.4	0.001
	83	26.6	19.5	-79.1 to 64.8	0.580
	28	31.5	4.7	-112.2 to 57.1	0.915

[a] The percent inhibition in oocyst density (%TRA), 95% confidence interval (95%CI) and p-values were calculated using a zero-inflated negative binomial (ZINB) model (K. Miura *et al,* Vaccine, 2016;34(35)4145)

[b] Total IgG purified from mice immunized with GST protein (Anti-GST total IgG) was used as an assay control to calculate %TRA of test samples (M. Tachibana *et al,* Vaccine, 2019;37(13)1799).

Supplementary Table 2: **SMFA data of original total IgGs (Table 1)**

IgG name	IgG Conc. [μg/mL]	Mean oocyst		%TRA		
		Feed 1	Feed 2	%TRA	95%CI	p-value
Set 1						
Control IgG	*1,500*	*31.0*	*15.5*			
cPro/CM1-4	1,500	1.3	1.2	94.5	89.6 to 97.6	0.001
cPro/CM1-3	1,500	0.0	0.0	100.0	99.4 to 100	0.001
cPro/CM1-2	1,500	4.3	0.4	94.0	88.6 to 97.3	0.001
cPro/CM1	1,500	0.3	0.4	98.6	97.1 to 99.5	0.001
Set 2						
Control IgG	*1,500*	*8.9*	*23.1*			
shPro/CM1	1,500	0.0	1.1	98.9	97.3 to 99.6	0.001

Supplementary Table 3: **SMFA data of cPro/CM1-2 depleted IgGs (Table 1)**

IgG name[a]	IgG Conc. [μg/mL]	Mean oocyst	%TRA		
			%TRA	95%CI	p-value
Feed 1					
Control IgG	*1,500*	*16.1*			
cPro/CM1-4	1,500	21.9	-35.5	-190.6 to 40.4	0.458
cPro/CM1-3	1,500	14.6	9.5	-102.5 to 62.0	0.813
cPro/CM1-2	1,500	14.0	13.5	-83.6 to 59.8	0.741
cPro/CM1	1,500	12.0	25.6	-55.4 to 65.1	0.436
shPro/CM1	1,500	21.9	-35.8	-186.8 to 37.5	0.436
Feed 2					
Control IgG	*1,500*	*74.4*			
GST	1,500	56.7	23.8	-62.0 to 64.8	0.492

[a] Name of total IgG from where region-specific antibodies were depleted is shown, except *control IgG.*

Supplementary Table 4: **SMFA data of shPro/CM1 depleted IgGs (Table 2)**

IgG name[a]	IgG Conc. [µg/mL]	Mean oocyst		%TRA		
		Feed 1	Feed 2	%TRA	95%CI	p-value
Control IgG	*1,500*	*23.0*	*32.9*			
cPro/CM1-4	1,500	7.3	17.9	58.4	27.4 to 76.8	0.004
cPro/CM1-3	1,500	5.7	10.5	71.9	52.6 to 84.5	0.001
cPro/CM1-2	1,500	5.6	6.9	77.6	59.4 to 87.8	0.001
cPro/CM1	1,500	9.7	21.2	48.1	6.1 to 70.1	0.032
shPro/CM1	1,500	3.5	10.5	78.0	61.6 to 88.1	0.001
GST	1,500	21.1	38.2	-3.3	-81.0 to 40.1	0.909

[a] Name of total IgG from where region-specific antibodies were depleted is shown, except *Control IgG*.

Supplementary Table 5: SMFA data of cPro/CM1-2-specific IgGs (Fig 3a)

IgG name[a]	IgG Conc[b] [EU]	Mean oocyst	%TRA		
			%TRA	95%CI	p-value
Feed 1[c]					
Control IgG	*N.A.*	*12.7*			
cPro/CM1-4	170,740	0.9	94.7	88.0 to 97.9	0.001
cPro/CM1-3	187,167	0.0	100	99.2 to 100	0.001
cPro/CM1-2	193,831	0.2	99.1	97.9 to 99.6	0.001
cPro/CM1	146,327	0.0	100	99.2 to 100	0.001
shPro/CM1	221,462	0.0	100	99.2 to 100	0.001
Feed 2					
Control IgG	*N.A.*	*22.9*			
cPro/CM1-4	20,000	13.2	42.6	-21.9 to 74.5	0.158
cPro/CM1-3	20,000	1.0	95.6	90.3 to 98.4	0.001
cPro/CM1-2	20,000	5.4	76.7	48.3 to 89.7	0.001
cPro/CM1	20,000	4.0	82.6	55.6 to 95.1	0.002
shPro/CM1	20,000	1.3	94.3	86.9 to 98.1	0.002

[a] Name of total IgG from where region-specific antibodies were purified is shown, except *Control IgG*.

[b] Anti-cPro/CM1-2 ELISA units.

[c] All cPro/CM1-2-specific IgGs were tested at the highest concentration possible. The data are not used in Fig 3a.

Supplementary Table 6: SMFA data of shPro/CM1-specific IgGs (Fig 3c)

IgG name[a]	IgG Conc[b] [EU]	Mean oocyst	%TRA		
			%TRA	95%CI	p-value
Control IgG	*N.A.*	*32.9*			
cPro/CM1-4	15,000	16.1	51.2	-10.3 to 78.4	0.096
cPro/CM1-3	15,000	1.5	95.6	89.5 to 98.1	0.001
cPro/CM1-2	15,000	3.2	90.3	77.0 to 96.0	0.001
cPro/CM1	15,000	8.0	75.8	47.0 to 89.2	0.001
shPro/CM1	15,000	7.2	78.1	48.9 to 90.6	0.001

[a] Name of total IgG from where region-specific antibodies were purified is shown, except *Control IgG.*

[b] Anti-shPro/CM1 ELISA units.

Supplementary Table 7: SMFA data of cPro-specific IgGs (Fig 4a)

IgG name[a]	IgG Conc. [μg/mL]	Mean oocyst	%TRA		
			%TRA	95%CI	p-value
Feed 1					
Control IgG	*750*	*18.0*			
cPro/CM1-4	303	0.3	98.6	96.1 to 99.8	0.001
cPro/CM1-3	315	0.1	99.4[b]	98.3 to 99.9	0.001
cPro/CM1-2	322	0.2	98.9	69.0 to 99.7	0.001
cPro/CM1	262	0.0	100.0[b]	99.3 to 100	0.001
shPro/CM1	151	0.1	99.4[b]	98.3 to 99.9	0.001
Feed 2					
Control IgG	*750*	*69.8*			
cPro/CM1-4	101	32.1	54.1	-0.7 to 78.5	0.055
	51	41.5	40.6	-27.8 to 73.6	0.174
	25	63.5	9.1	-94.1 to 57.6	0.811
cPro/CM1-3	105	0.4	99.5[b]	98.9 to 99.8	0.001
	53	7.1	89.8	77.6 to 95.8	0.001
	26	37.8	45.9	-15.6 to 74.8	0.123
cPro/CM1-2	107	5.4	92.3	83.0 to 96.5	0.001
	54	31.7	54.6	7.0 to 78.5	0.033
	27	51.2	26.7	-54.6 to 66.9	0.423
cPro/CM1	87	3.0	95.7	90.5 to 98.3	0.001
	44	30.5	56.3	4.4 to 79.6	0.042
	22	56.0	19.8	-66.4 to 64.0	0.527
shPro/CM1	50	24.3	65.2	26.4 to 83.6	0.007
	25	37.3	46.6	-14.9 to 75.2	0.110
	13	62.7	10.2	-96,3 to 60.7	0.754
Feed 3					
Control IgG	*750*	*25.0*			
cPro/CM1-4	59	2.0	92.2	81.6 to 97.2	0.001
cPro/CM1-3	62	1.0	96.0	90.6 to 98.6	0.001
cPro/CM1-2	64	3.0	88.0	72.9 to 95.2	0.001
cPro/CM1	65	2.0	92.2	80.6 to 97.7	0.001
shPro/CM1	56	1.1	95.8	90.7 to 98.2	0.001

[a] Name of total IgG from where region-specific antibodies were purified is shown, except *Control IgG.*

[b] The data were not used for IC_{80} calculations, as the %TRA values were at the top-plateau part of dose-responses.

Supplementary Table 8: SMFA data of shPro-specific IgGs (Fig 4b)

IgG name[a]	IgG Conc. [μg/mL]	Mean oocyst	%TRA		
			%TRA	95%CI	p-value
Feed 1					
Control IgG	*750*	*15.5*			
cPro/CM1-4	132	5.1	67.4	2.7 to 87.6	0.013
cPro/CM1-3	396	0.0	100.0[b]	98.4 to 100	0.001
cPro/CM1-2	216	0.1	99.4[b]	98.0 to 99.9	0.001
cPro/CM1	289	0.2	99.0[b]	97.3 to 99.8	0.001
shPro/CM1	698	0.1	99.4[b]	97.6 to 99.8	0.001
Feed 2					
Control IgG	*750*	*20.6*			
cPro/CM1-3	66	0.6	97.1	93.2 to 98.9	0.001
	33	10.2	50.6	-8.9 to 78.5	0.078
	17	25.5	-23.8	-183.8 to 46.2	0.600
cPro/CM1-2	36	8.2	60.3	11.9 to 82.2	0.032
	18	18.6	9.7	-99.5 to 61.1	0.802
	9	21.3	-3.4	-124.2 to 54.0	0.958
cPro/CM1	48	0.5	97.8	93.4 to 99.7	0.001
	24	8.3	59.6	9.1 to 82.4	0.030
	12	15.2	26.3	-64.5 to 67.4	0.480
shPro/CM1	116	0.8	96.4	90.6 to 99.1	0.001
	58	7.5	63.5	14.5 to 83.9	0.017
	29	20.6	-0.2	-132.5 to 55.6	0.971
Feed 3					
Control IgG	*750*	*74.4*			
cPro/CM1-3	51	6.6	91.1	80.6 to 96.4	0.001
cPro/CM1-2	64	10.5	85.9	69.6 to 93.9	0.001
cPro/CM1	53	17.2	76.9	51.9 to 89.1	0.001
shPro/CM1	62	20.6	72.4	42.5 to 87.3	0.003

[a] Name of total IgG from where region-specific antibodies were purified is shown, except *Control IgG.*

[b] The data were not used for IC_{80} calculations, as the %TRA values were at the top-plateau part of dose-responses.

Supplementary Table 9: SMFA data for Fig 5e[a]

IgG name	IgG Conc. [µg/mL]	Mean oocyst	%TRA		
			%TRA	95%CI	p-value
Feed 1					
with complement					
Control IgG	*1,500*	*22.9*			
shPro-KLH	1,500	0.2	99.3	98.3 to 99.8	0001
KLH	1,500	22.5	1.9	-110.2 to 55.3	0.946
Feed 2					
with complement					
Control IgG	*1,500*	*74.4*			
shPro-KLH	1,500	0.2	99.8	99.5 to 99.9	0.001
	375	18.0	75.9	48.7 to 89.4	0.002
	94	99.3	-33.4	-179.5 to 40.2	0.477
	23	42.2	43.3	-20.8 to 73.6	0.139
KLH	1,500	78.5	-5.5	-121.5 to 53.6	0.895
Feed 3					
with complement					
Control IgG	*1,500*	*29.4*			
shPro-KLH	1,500	0.9	97.1	93.6 to 98.8	0.001
	375	8.9	69.9	39.2 to 85.9	0.002
	94	35.4	-20.3	-165.0 to 45.3	0.677
KLH	1,500	20.7	29.5	-59.4 to 68.5	0.384
without complement					
shPro-KLH	1,500	25.8	12.2	-90.2 to 60.2	0.733
	375	49.4	-68.2	-261.9 to 23.5	0.196
	94	49.3	-67.8	-280.7 to 21.3	0.188
KLH	1,500	31.6	-7.4	-127.1 to 48.7	0.864

[a] All SMFAs were performed with human complement (i.e., with 31% v/v of non-heat inactivated human serum), except Feed 3 where both with and without complement conditions were evaluated.

Supplementary Table 10: **SMFA data for Fig 6e**[a]

IgG name	IgG Conc. [µg/mL]	Mean oocyst		%TRA[b]		
		Feed 1	Feed 2	%TRA	95%CI	p-value
Control IgG	*1,500*	*12.8*	*69.2*			
0.2 µg dose with CP	1,500	6.9	45.4	40.4	-2.4 to 66.0	0.057
2 µg dose with CP	1,500	3.7	28.1	65.9	41.1 to 80.8	0.001
0.2 µg dose with CPQ	1,500	0.7	11.0	90.7	83.4 to 95.2	0.001
2 µg dose with CPQ	1,500	0.0	0.1	99.8	99.4 to 99.9	0.001
CPQ alone[c]	1,500		52.4	24.2	-64.5 to 65.2	0.490

[a] Both SMFAs were performed with human complement (i.e., with 31% v/v of non-heat inactivated human serum).

[b] The %TRA, 95%CI and p-values from two feeds, except for CPQ alone group, which was tested one feed (Feed 2).

[c] The mouse sera were generated in a different immunization study, but using the same immunization regimen. The purified IgG was tested only in Feed 2.

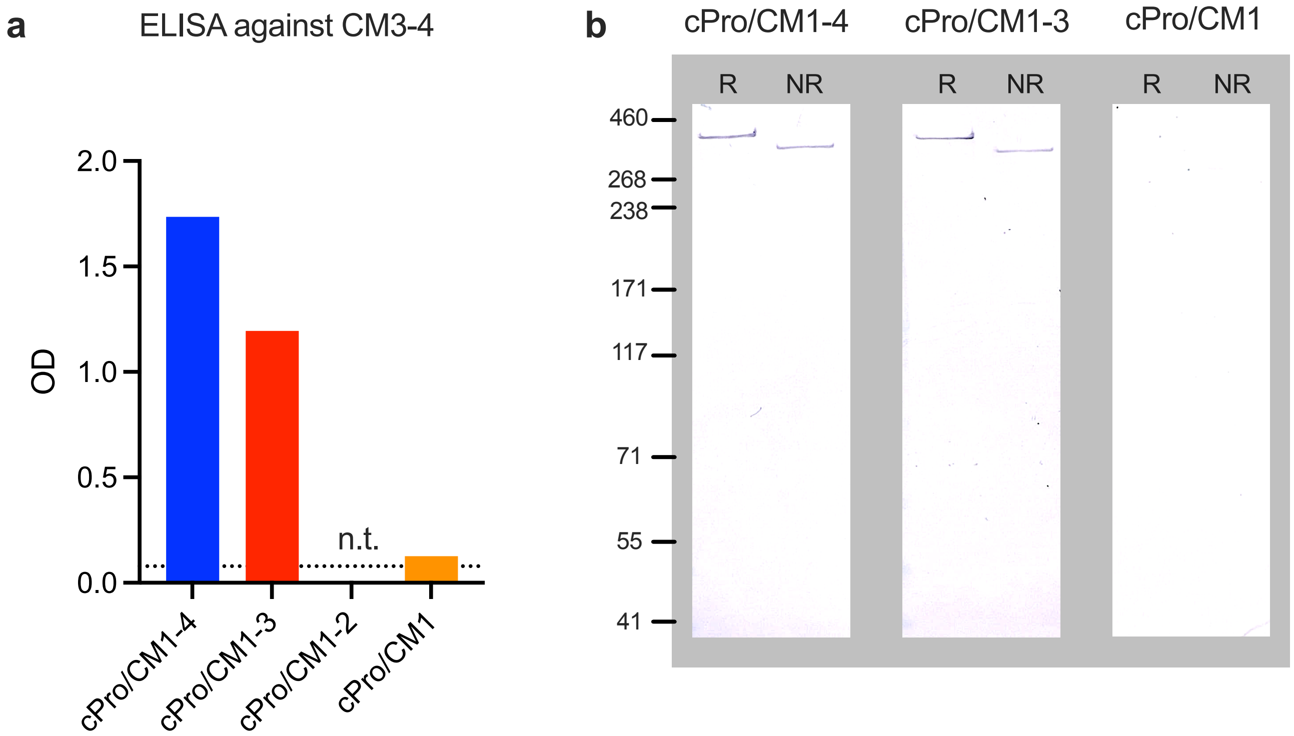

Supplementary Figure 1: **ELISA and western blot for cPro/CM1-2 depleted IgGs .** From the original total IgGs, cPro/CM1-2-specific antibodies were depleted. The ELISA and SMFA results are shown in Table 1. (**a**) The depleted IgGs were tested at 3 μg/mL against a CM3-4 recombinant protein by ELISA. Instead of ELISA units, OD values are shown. The depleted IgG from anti-cPro/CM1-2 total IgG was not tested (n.t.), as no IgG was available. The dotted line indicates average OD value in blank wells. (**b**) Reactivity of the depleted IgGs against native Pfs230 was assessed by western blottting.

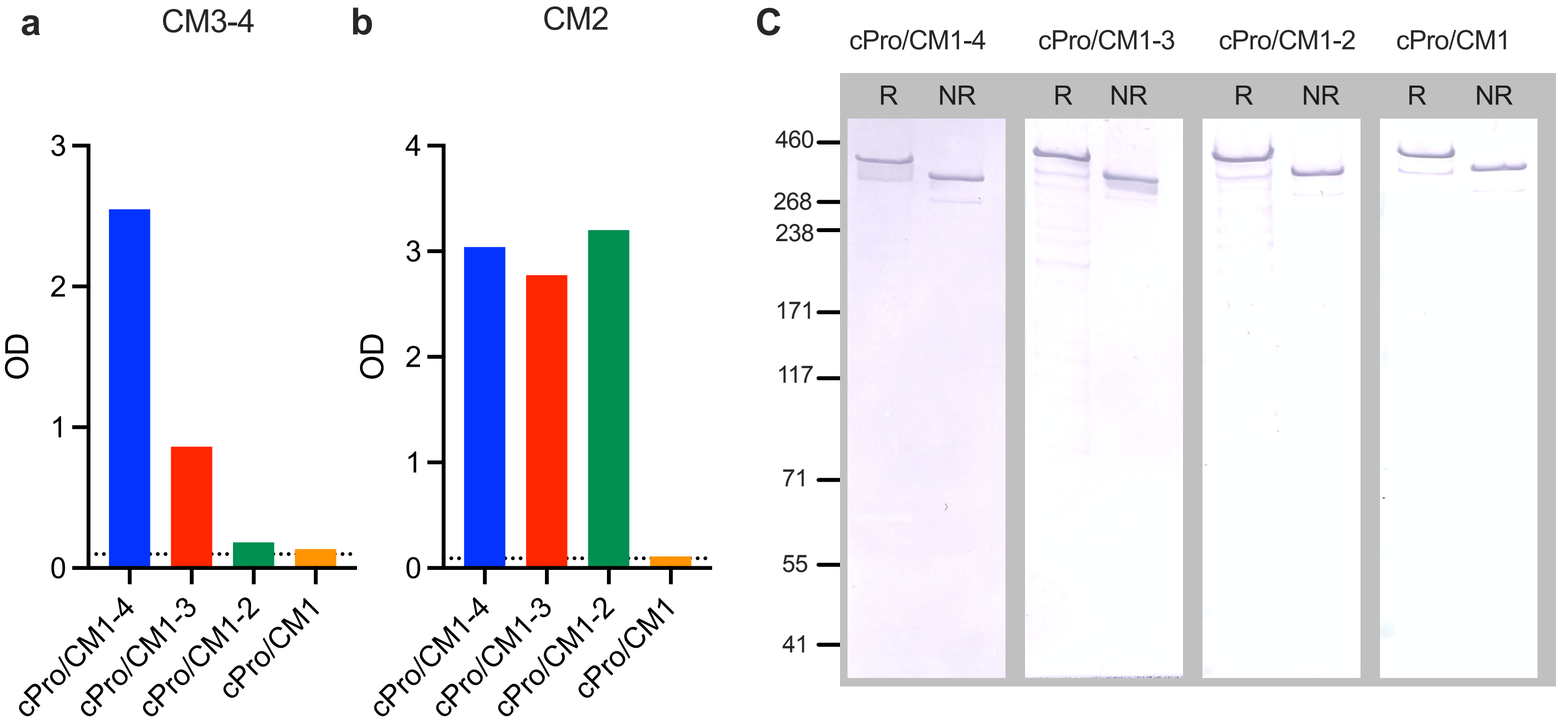

Supplementary Figure 2: **ELISA and western blot for shPro/CM1 depleted IgGs .** From the original total IgGs, shPro/CM1-specific antibodies were depleted, and the results are shown in Table 2. The depleted IgGs were tested at 3 µg/mL against CM3-4 (**a**) or CM2 (**b**) recombinant proteins by ELISA. Instead of ELISA units, OD values are shown. The dotted lines indicate average OD values in blank wells. (**c**) Reactivity of the depleted IgGs against native Pfs230 was assessed by western blottting.

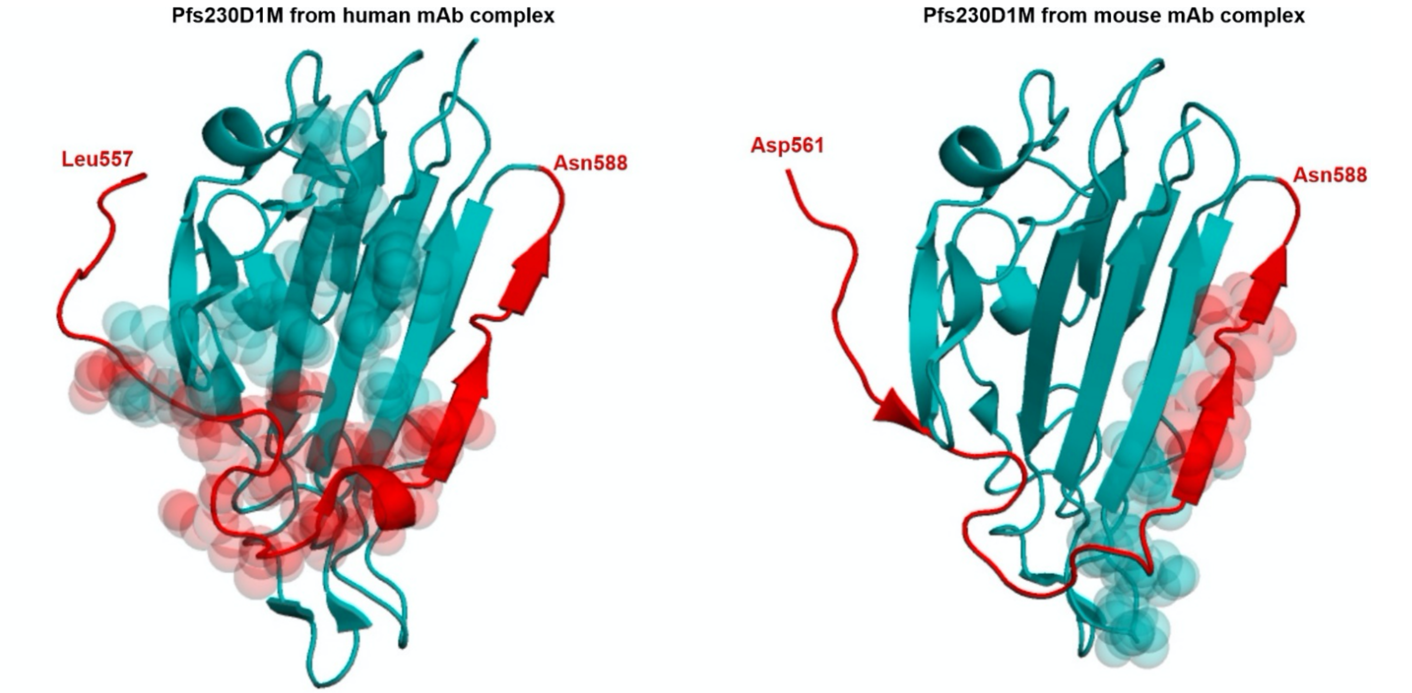

Supplementary Figure S3: Structure of shPro within Pfs230D1M, complexed with transmission blocking antibodies. Pfs230D1M (aa 542-736) in complex with human mAb LMIV230-01 (PDB-ID, 7JUM) or mouse mAb 4F12 (PDB-ID, 6OHG) is shown. All residues of Pfs230D1M that make contact with these mAbs are indicated as semi-transparent spheres. The shPro portion of Pfs230D1M (and the bounding residues of shPro) are labeled in red, while the remainder of the protein is shown in teal. Within Pfs230D1M (aa 542-736) or shPro (aa 543-588), the N-terminal portion (before aa 557) lacks structural information. To assess the structure, pdb files 7JUM (C. Coelho *et al*, Nat Commum, 2021;12;1750) and 6OHG (K. Singh *et al*, Commun Biol, 2020;3;395) were downloaded from the RCSB protein data bank and visualized with Pymol software.

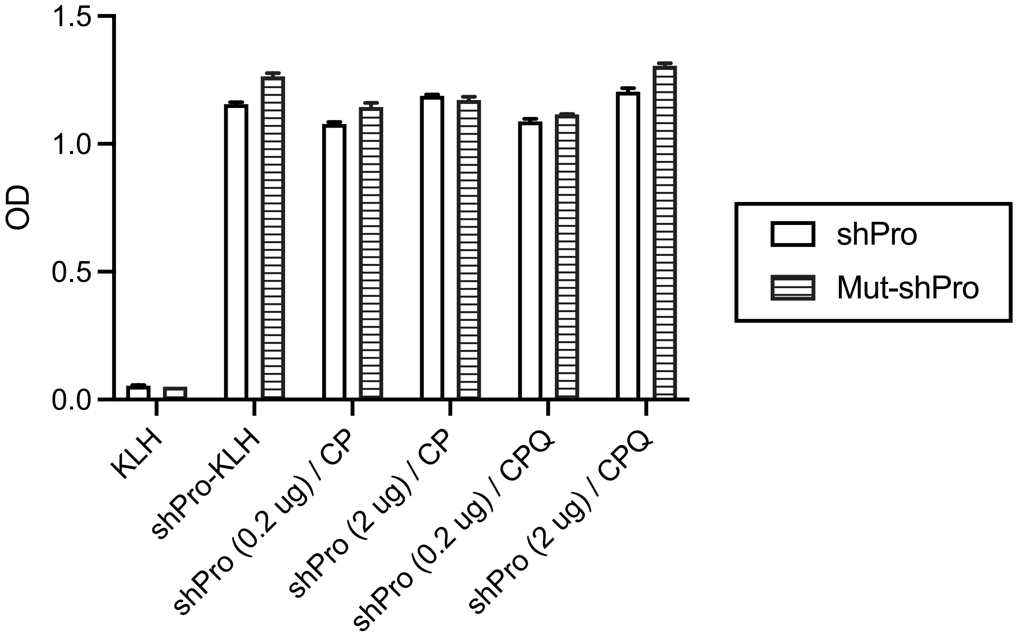

Supplementary Figure 4: **ELISA against original shPro and mutant shPro peptides.** Total IgGs used for SMFA in Fig 5e (anti-shPro-KLH total IgG) and Fig 6e (4 total IgGs) were diluted at 1 anti-shPro ELISA unit, and tested against the original shPro and a mutant shPro (Mut-shPro) peptides. The Mut-shPro peptide involved changing the 9th amin acid from proline to serine. As a negative control, anti-KLH total IgG was tested at the same protein concentration as anti-shPro-KLH total IgG. Mean (and standard deviation) OD values in triplicate wells are shown.

a

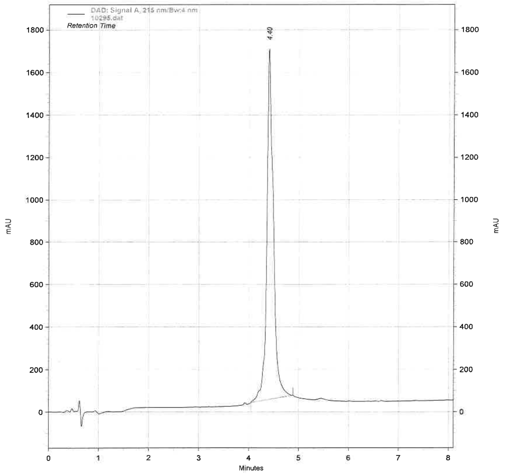

b

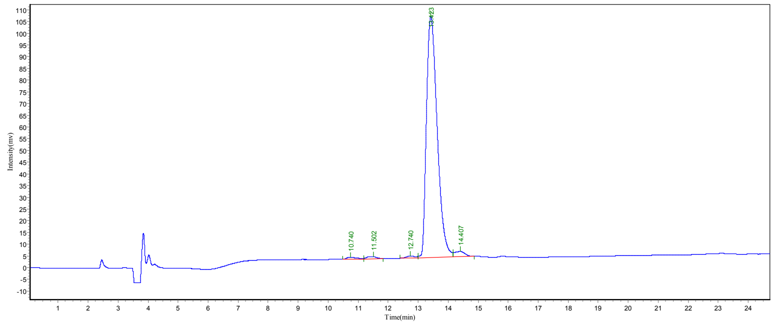

Supplementary Figure S5: HPLC analysis for shPro peptides. The peptides made by RTB/NIAID (**a**) and WatsonBio Sciences (**b**). Based on the main peak areas, the purity was calculated as >99.9% (**a**) and 95.1% (**b**), respectively.

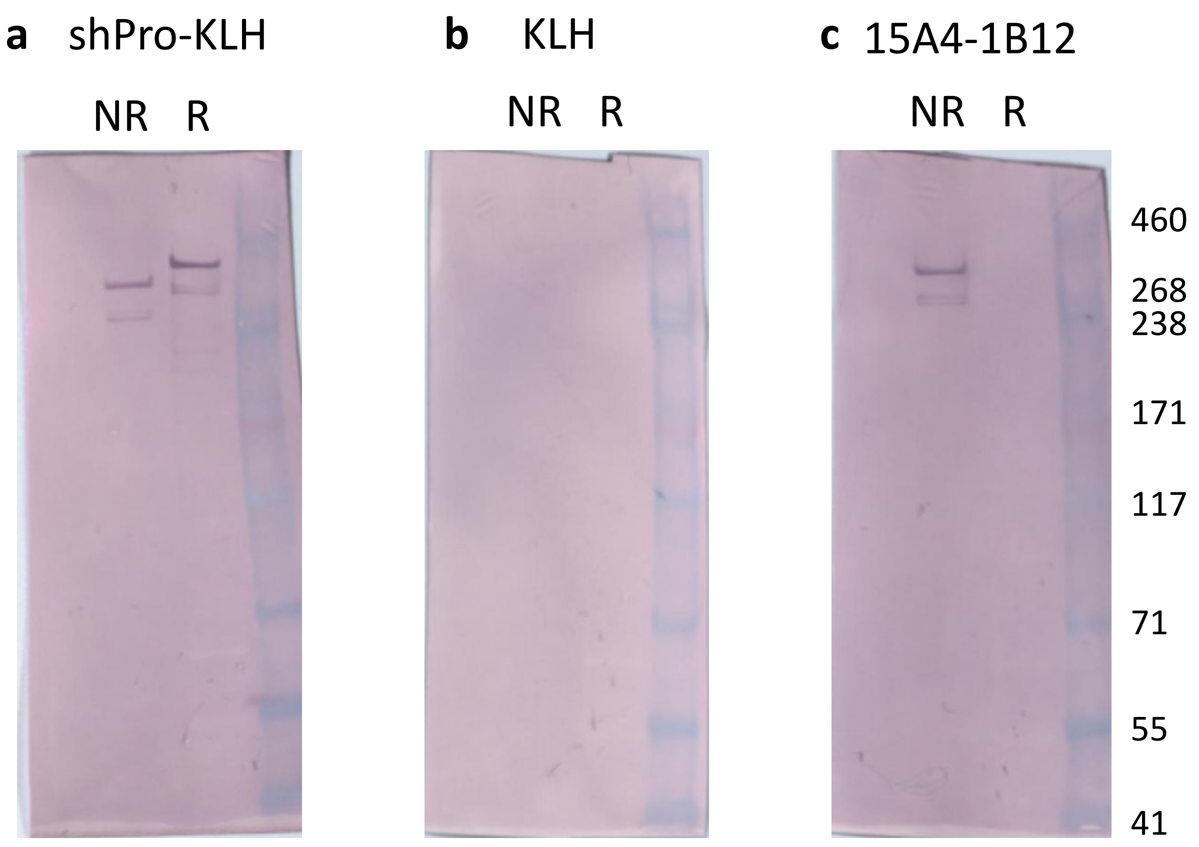

Supplementary Figure S6: Uncropped western blot images for Fig 5d. Reactivity of purified total IgG from each group against native Pfs230 was assessed by western blotting. Gametocyte extracts were tested under reducing (R) and non-reducing (NR) conditions. The conformation-dependent mouse anti-Pfs230C1 (aa 443-731) monoclonal antibody, 15A4-1B12, was used as a positive control.
